# Collective Trauma and the Social Construction of Meaning

**DOI:** 10.3389/fpsyg.2018.01441

**Published:** 2018-08-10

**Authors:** Gilad Hirschberger

**Affiliations:** Interdisciplinary Center Herzliya, Herzliya, Israel

**Keywords:** collective trauma, meaning, social identity, victimization, collective memory

## Abstract

Collective trauma is a cataclysmic event that shatters the basic fabric of society. Aside from the horrific loss of life, collective trauma is also a crisis of meaning. The current paper systematically delineates the process that begins with a collective trauma, transforms into a collective memory, and culminates in a system of meaning that allows groups to redefine who they are and where they are going. For victims, the memory of trauma may be adaptive for group survival, but also elevates existential threat, which prompts a search for meaning, and the construction of a *trans*-generational collective self. For perpetrators, the memory of trauma poses a threat to collective identity that may be addressed by denying history, minimizing culpability for wrongdoing, transforming the memory of the event, closing the door on history, or accepting responsibility. The acknowledgment of responsibility often comes with disidentification from the group. The dissonance between historical crimes and the need to uphold a positive image of the group may be resolved, however, in another manner; it may prompt the creation of a new group narrative that acknowledges the crime and uses it as a backdrop to accentuate the current positive actions of the group. For both victims and perpetrators, deriving meaning from trauma is an ongoing process that is continuously negotiated within groups and between groups; it is responsible for debates over memory, but also holds the promise of providing a basis for intergroup understanding.

## Introduction

The term *collective trauma* refers to the psychological reactions to a traumatic event that affect an entire society; it does not merely reflect an historical fact, the recollection of a terrible event that happened to a group of people. It suggests that the tragedy is represented in the collective memory of the group, and like all forms of memory it comprises not only a reproduction of the events, but also an ongoing reconstruction of the trauma in an attempt to make sense of it. Collective memory of trauma is different from individual memory because collective memory persists beyond the lives of the direct survivors of the events, and is remembered by group members that may be far removed from the traumatic events in time and space. These subsequent generations of trauma survivors, that never witnessed the actual events, may remember the events differently than the direct survivors, and then the construction of these past events may take different shape and form from generation to generation. Such collective memory of a calamity suffered in the past by a group’s ancestors may give rise to a *chosen trauma* dynamic that weaves the connection between trauma, memory and ontological security (Volkan, 1997). These chosen traumas are conceptualized as narratives emphasizing that ‘walking through blood’ is necessary on the path to freedom, independence and group security (Resende and Budryte, 2014). In this paper I illustrate how the collective memory of traumatic events is a dynamic social psychological process that is primarily dedicated to the construction of meaning. The creation and maintenance of meaning comprises a sense of self-continuity, a connection between the self, others and the environment (Baumeister and Vohs, 2002; Heine et al., 2006), and the feeling that one’s existence matters. It is a processes of identity construction that comprises the sense of self-esteem, continuity, distinctiveness, belonging, efficacy, and ultimately a sense of meaning (Vignoles et al., 2006). Accordingly, the current article relies on these principles to trace the process of meaning-making following historical trauma at the collective level and among both victim and perpetrator groups.

Much of the theory and research presented in this paper focuses on the Holocaust because it is considered to be the prototypical 20th century genocide, and has attracted more attention and scholarship than other collective traumas (Mazur and Vollhardt, 2015). Can the Holocaust be compared to other cases of genocide and mass murder and should it? According to eminent Holocaust historian, Yehuda Bauer, the Holocaust, in spite of its unique attributes, can and must be compared to other events of a similar nature, otherwise why should a public school system in Philadelphia, New York, or Timbuktu teach it (Bauer, 1979)? Based on the notion that every specific trauma is unique, but the lessons derived can be universal, this paper discusses the common long-term consequences of different forms of collective victimization.

For victims of collective trauma meaning is established by: (a) passing down culturally-derived teachings and traditions about threat that promote group preservation; (b) these traditions of threat amplify existential concerns and increase the motivation to embed the trauma into a symbolic system of meaning; (c) trauma fosters the sense of a collective self that is transgenerational thereby promoting a sense of meaning and mitigating existential threat; (d) the sense of an historic collective self also increases group cohesion and group identification that function to create meaning and alleviate existential concerns; (e) the profound sense of meaning that is borne out of collective trauma perpetuates the memory of the trauma and the reluctance to close the door on the past; (f) Over time collective trauma becomes the epicenter of group identity, and the lens through which group members understand their social environment.

For members of perpetrator groups, collective trauma represents an *identity threat* (Branscombe et al., 1999), as it creates tension between the desire to view the group in a positive light (Tajfel and Turner, 1979), and the acknowledgment of severe moral transgressions in its past. The inability to reconcile the character of the group in the present with its character in the past may motivate group members, primarily high identifiers, to perceive an historical discontinuity of the group that serves to distance present group members from past offenders (Roth et al., 2017). Sometimes this discontinuity is reflected in the motivation to close the door on history and never look back (Imhoff et al., 2017), and sometimes the thorny chapters of a group’s history are glossed over creating an uncomfortable gap in collective memory – an absence suggesting a presence. Members of perpetrator groups may deal with the dark chapter in their history by thoroughly denying the events, disowning them and refusing to take any responsibility for them. But, more often than not, reactions to an uncomfortable history will take on a more nuanced form with group members reconstructing the trauma in a manner that is more palatable, and representing the trauma in a manner that reduces collective responsibility. In some cases, the dissonance between current group values and past behavior are so great that disaffiliation from the group remains the only viable option (Čehajić and Brown, 2010; Hirschberger et al., 2016b).

Understanding the impact of trauma on collective meaning becomes even more complex when considering what Primo Levi defined as the *gray zone* (Levi, 1959) – a nebulous area wherein the distinction between victims and perpetrators is not always clear cut, and victims may behave as perpetrators and perpetrators are victims. Members of groups that exist in this region of collective memory are often motivated to defensively represent their history in a manner that highlights their sacrifice and downplays their crimes (Bilewicz et al., 2014; Hirschberger et al., 2016b). These groups may also engage in competitive victimhood dynamics with other groups demanding to be recognized as the veritable victim (Noor et al., 2012). Sometimes the victimization of one group poses such a threat to another unrelated victimized group because of the sense that its’ victimization is overshadowed and does not receive due attention and acknowledgment. For example, sub-Saharan African immigrants in Belgium who felt a lack of recognition of their group’s victimization expressed more antisemitism as a form of competitive victimhood with Jews whose victimization receives more recognition (De Guissmé and Licata, 2017). The current paper offers a perspective suggesting that the intra- and inter-group tribulations over a dark chapter in history represent more than an attempt to abdicate responsibility for past crimes, or quarrel over the benefits of the victim status. The need to come to terms with a dark past represents a crisis of meaning that must be resolved for the group to deconstruct and reconstruct its sense of collective self and assume an identity that offers continuity, coherence and significance. The memory of historical crimes threatens fundamental values, current notions of self-worth, and the sense of having a constructive collective purpose (Baumeister, 1991; Vignoles et al., 2006). The quest for meaning must, therefore, involve the reconstruction of these basic elements.

This analysis of meaning borne out of trauma for both victim and perpetrator groups offers the provocative suggestion that trauma is not merely a destructive event, but also an irreplaceable ingredient in the construction of collective meaning. Accordingly, for victim groups there may be secondary gains to collective trauma, that are often overlooked, that function to keep the memory of trauma alive, and lead subsequent generations to incorporate the trauma into their collective self. For perpetrator groups, the trauma functions as a catalyst that stimulates the construction of a new social representation that, if successful, can support a collective self that acknowledges past transgressions in a manner that is neither defensive nor crippling; one that promotes positive social identity (e.g., Vignoles et al., 2006) predicated on the triumph over past failings. On this basis, the present article considers alternative ways to remember collective trauma that can break out of compulsive reenactments of the past, or defensive dynamics; ways that may reconcile the *meaning wars* between groups with a convoluted history and reduce intergroup tension and hostility.

## From Disintegration to Newfound Meaning

Collective trauma is devastating for individuals and for groups; it constitutes a cataclysmic event that affects not only direct victims, but society as a whole. Just as trauma at the individual level shatters assumptive worldviews about oneself and one’s position in the world (Janoff-Bulman, 1992), so does collective trauma transform the way survivors perceive the world and understand the relationship between their group and other groups, even those unrelated to the initial victimization (Alexander et al., 2004; Vollhardt, 2012). Trauma can disrupt people’s global sense of meaning by exposing them to the darker sides of human nature (Park, 2013). Establishing meaning, therefore, is particularly important when individuals (or groups) encounter traumatic life experiences (Park, 2013). Sociologist Kai Erikson eloquently describes the similarities and differences between individual and collective trauma and their impact on the self:

“*by individual trauma I mean a blow to the psyche that breaks through one’s defenses so suddenly and with such brutal force that one cannot react to it effectively…by collective trauma, on the other hand, I mean a blow to the basic tissues of social life that damages the bonds attaching people together and impairs the prevailing sense of communality. The collective trauma works its way slowly and even insidiously into the awareness of those who suffer from it, so it…[is] a gradual realization that the community no longer exists as an effective source of support and that an important part of the self has disappeared… ‘We’ no longer exist as a connected pair or as linked cells in a larger communal body”* (Erikson, 1976, pp. 153–154).

Erikson’s depiction of the disintegration of social support systems in the face of collective trauma clarifies the crisis of meaning that ensues and echoes Baumeister and Vohs’ (2002, p. 608) contention that “the essence of meaning is connection.” Collective trauma undermines a fundamental sense of security with long-standing effects among second and third generations of survivors. At the personal level, these individuals display significantly higher rates of psychological distress (Yehuda et al., 2002); at the social level second and third generation survivors display heightened individual and collective fear, feelings of vulnerability, injured national pride, humiliation (Lifton, 2005), a crisis of identity, and a predisposition to react with heightened vigilance to new threats, such that the pain of past generations is conflated with threats facing the current generation (Canetti et al., 2018).

The catastrophic image of the long-lasting effects of collective trauma portrayed by Erikson, albeit valid, represents only one side of the coin on how traumatic historical events impact individuals and groups. The current paper places the spotlight on another important aspect of collective trauma that has received less attention – the relationship between collective trauma and the construction of meaning. Although trauma is undoubtedly destructive, meaning is often unexpectedly found in calamity (Frankl, 1959/1976) and facilitated by processes of sense making (Davis et al., 1998). Trauma may contribute to the creation of a national narrative (Alexander et al., 2004), a sense of identity (Canetti et al., 2018), and cognitive working models that ostensibly function to ensure the safety and well-being of the group and provide it with values and guidelines for the future (Bar-Tal and Antebi, 1992; Hirschberger et al., 2017). Collective trauma may, therefore, facilitate the construction of the various elements of meaning and social identity: purpose, values, efficacy, and collective worth (Vignoles et al., 2006). These effects of trauma on the construction of collective meaning may, ironically, increase as time elapses from the traumatic event (Klar et al., 2013) because the focus of memory shifts from the painful loss of lives to the long-term lessons groups derive from the trauma.

The effects of collective trauma on the construction of meaning is not limited to the victim group that needs to reinvent itself and reconstruct all that was lost, but also to the perpetrator group that must redefine itself and construct a positive moral image of the group in light of the atrocities it committed (Shnabel and Nadler, 2008; Hirschberger et al., 2016b; Imhoff et al., 2017). The current paper traces the process of meaning-making for historical victims and perpetrators and suggests that although there are some pathological aspects to meaning borne out of trauma, these meaning structures ultimately contribute to group identification and cohesion, provide a sense of history and destiny, and propel groups to turn the calamity into a springboard for growth.

## Trauma Generates a Search for Meaning

Individuals and nations possess a collective memory (Halbwachs, 1980) of historical events, even those that took place long before they were born (Licata and Mercy, 2015). This collective memory does not constitute an accurate record of history, but rather is constructed by members of the group who function as ‘lay historians’ (Klein, 2013) in an attempt to inject meaning into history and provide a *usable past* (Wertsch, 2002; Licata and Mercy, 2015) that serves an important function in the present. One primary function of collective memories is to create and maintain social identity: “history provides us with narratives that tell us who we are, where we came from, and where we should be going. It defines a trajectory which helps construct the essence of a group’s identity” (Liu and Hilton, 2005, p. 537). Collective memory not only promotes the construction of identity, but also the preservation of a positive collective identity (Tajfel and Turner, 1979) and a sense of worth (Vignoles et al., 2006). This can be achieved through social comparisons and devaluations of other groups, and also through the reconstruction of reality and memory as to uphold a positive image of the group.

Collective trauma may threaten collective identity; it may raise questions about the significance of the group, and about core belief systems for both victims (e.g., “where was God when the trauma happened?”), and perpetrators (“How could my people commit such crimes?”); it may raise questions about the wisdom of continuing one’s affiliation with a victimized group because being a member could be physically dangerous, and may also include feelings of humiliation and loss of agency (Shnabel and Nadler, 2008). It may also threaten affiliation with perpetrator groups as members inevitably contend with a burden of guilt. These processes may compromise group cohesion and lead to the disintegration of the group. Collective trauma, however, does not necessarily have a negative impact on group identity and cohesion and often bolsters affiliation with the group through a feeling of shared fate and destiny – an integration of the traumatic experience into one’s identity and narrative (Gillies and Neimeyer, 2006). For instance, massacres and military defeats, as terrible as they may be, provide fertile ground for the production of cultural narratives and shared belief systems that infuse meaning and support social identity in the aftermath of calamity (Weber, 1946; Olick et al., 2011; László, 2013). Thus, historical trauma may be integrated into the social representation of both victim and perpetrator groups (i.e., “we are historical victims that continue to survive against all odds”; “it is our responsibility to promote values of acceptance and tolerance”), and then the trauma may have a solidifying and identity building effect as it becomes a central feature in collective memory and group narrative (Bar-Tal et al., 2009).

## Part I: Victims

### Why Do Victims Want to Remember?

The historical memory for collective trauma may span millennia, with groups commemorating traumatic events that can be traced back to antiquity, and even to biblical times. Muslims remember their battle with the crusaders at the Horns of Hattin; Jews are commanded to never forget Amalek – the biblical people who threatened the Israelites. More recently, the Irish commemorate the rebellions against the British; Koreans carry with them the scars of Japanese oppressive rule; Bosnians can never erase the atrocities of Srebrenica; and the legacy of the Holocaust is to never forget. These memories of victimization that may convey an unflattering image of group weakness and powerlessness (Shnabel et al., 2009; Vollhardt, 2012) raise the question: why do these people and many others cling to their traumatic memory as a cherished possession? Why do they not want to move on and let bygones be bygones? In the following sections the manner by which the painful memory of trauma is adaptive to individuals and groups is presented layer by layer. In the first layer, the basic evolutionary level, the memory of trauma is shown to promote vigilance that may enhance actual group survival and restore a sense of efficacy. The memory of trauma, however, serves the needs of individuals and groups far beyond its contribution to survival; the memory of trauma and the existential threat that is inherent to it motivate a desire to construct meaning around the experience of extreme adversity. In this process of meaning-making, a transgenerational collective self is pieced together – a self-transcendent historical identity that provides a sense of continuity between past, present and future members of the group (Kahn et al., 2017). This transgenerational collective self promotes group cohesion, a sense of group importance and common destiny, and a strong commitment to group identity. This aspect of trauma reestablishes a sense of control, bolsters self/collective worth and prompts the search for meaning in suffering. To let go of the trauma, is therefore, highly aversive and costly; it is akin to abdicating collective meaning; and against this threat to meaning societies mobilize to keep the trauma alive as a lesson from the past to the future.

### Traumatic Memory Is Adaptive

The 2004 Indian Ocean tsunami caused great devastation and a tragically high mortality rate – up to 90% of the population dead in some locations. In 1930, a tsunami of similar magnitude struck Papua New Guinea with only a fraction of that death toll – less than 1% of the population perished. According to a study on cultural responses to tsunamis (Mercer et al., 2012), this curious discrepancy in the lethality of two similar natural disasters can be attributed to a seemingly implausible cause: oral traditions. These traditions passed down from generation to generation over hundreds of years included, in the case of Papuan culture, the unequivocal instruction to run for the hills when the sea draws down. Indeed, Papuans who did not question this tradition, successfully escaped an almost certain death. An analysis of the communities that were most hard-hit in the 2004 tsunami reveal that these were mostly recent immigrants to coastal regions that had no collective memory about tsunamis, and no tradition on how to identify this threat and defend against it (Mercer et al., 2012). This comparison of two tsunamis provides a glimpse into how the memory of collective trauma (or the lack thereof) may directly influence group survival by promoting life-saving efficacy.

The collective memory of natural disasters and the collective memory of traumas intentionally caused by humans have much in common – they serve as guides for future generations on how to identify threat and how to respond to it effectively. However, whereas tsunamis will always be tsunamis with the lessons of the past forever applying to future generations, human societies change and evolve such that the villains of the past may have transformed and changed their relationship with the victim group. In this case, should the lessons of the past still inform future generations? From an evolutionary standpoint, exercising extra vigilance is warranted when it is not certain that the leopard has indeed changed its spots, or when this ostensible change is circumspect and may seem disingenuous. It makes good sense for victimized groups to keep their guard up, approach their past tormentors with some trepidation and hesitance, and ensure that future generations understand and remember the potential for danger.

Korean-Japanese relations, for instance, are still marked by Korean trepidation of their neighbor on the other side of the Tsushima Strait (Holmes, 2015). Although Japan has become a peaceful, even pacifist, country in the past 70 years since WWII, with one of the smallest military expenditures per GDP in the world, its record of aggression against Korea stretches back to the 16th century. Koreans are, therefore, still weary of their former occupier, and keep their guard up to the possibility that Tokyo may someday revert back to its aggressive imperial past. This diffidence may not only reflect Korean frustration over the recent Japanese apology for sex slavery that many feel was disingenuous; it may reflect a gut instinct to steer clear of a group that caused them much harm in the past. This lack of historical closure that many Koreans feel with regards to Japan is often perceived as maladaptive because it stands in the way of intergroup harmony. But if the safety of the group is the ultimate goal, and intergroup relations are but a means toward this end, it makes clear sense to distrust and remain vigilant toward a former adversary.

Similarly, Germany has undergone significant transformation and a conscious effort to sever any continuity between Germany today and the Third Reich (Hein and Selden, 2000). Many of Germany’s neighbors such as France (Hanke et al., 2013) and Poland (Imhoff et al., 2017) seem to recognize this transformation, and are able to separate the Germany of the past from that of the present. Israeli Jews, however, show a more ambivalent reaction, and a greater reluctance to close the book on the Holocaust and achieve closure; they are also more likely to conflate the past with the present, such that their attitudes toward contemporary Germany are contingent on their attributions for the past (Imhoff et al., 2017). Because the Holocaust is but the tragic climax of centuries of German and European anti-Semitism, many Jews are reluctant to let go of the past, and when engaging with contemporary Germans even on issues unrelated to the past, the Holocaust is often implicitly present (Imhoff, 2009).

The motivation to perceive continuity between the historical perpetrator group and current group members reflects ongoing caution toward a group that is still perceived as potentially dangerous. For instance Lebanese Maronite Christians who identify with their group perceived greater continuity among current and past members of their former enemies, Lebanese Muslims (Licata et al., 2012), indicating that they were motivated to view the current group as potentially having the same malevolent intents as the historical group.

These collective reactions to a history of trauma are similar in many respects to individual post-traumatic reactions. The experience of trauma at the individual level may lead to a post-traumatic reaction characterized by hypervigilance, re-experiencing the event, and avoidance of stimuli that are reminiscent of the event (Solomon and Mikulincer, 2006). Although such reactions may be debilitating, there are also adaptive elements to this extreme response that should not be ignored. A near-death experience often teaches people that greater vigilance and attention to threat are warranted to avoid the recurrence of such a life-threatening situation.

This dynamic of once bitten twice shy can be explained at the very basic evolutionary level as fear conditioning – an adaptive response to a threatening stimulus that is easily acquired, but is highly resistant to change (LeDoux, 1996). It can be inferred, therefore, that the same mechanisms that keep individuals out of harm’s way, operate to safeguard group survival by maintaining heightened and prolonged vigilance toward out-groups that posed a threat to the group in the past.

### From Adaptive Vigilance to Post-traumatic Worldviews

This relatively straightforward evolutionary explanation, however, does not suffice to explain the adaptive function of keeping the memory of trauma alive, because in some cases the historical perpetrator is no longer present. In these cases, an evolutionary explanation of vigilance in the face of a potentially dangerous adversary does not hold. One other way to explain the cultivation of an historical memory, that still remains within an evolutionary framework, is that vigilance born of trauma does not have to be directed toward a specific perpetrator group and can be generalized into a chronic and diffuse vigilance toward all other groups. Just as little Albert learned to generalize his fear to all furry objects in ((s))Watson and Rayner (1920) cruel experiment, groups may learn that members of other groups harbor animosity toward them, and that the perpetrator only changes face, not harmful intent. This generalization of fear may reflect a harsh recognition that the group is, in fact, a target of hate by many other groups, and then an expectation for mistreatment by other group members seems reasonable.

For instance, the long history of persecution against the Jewish people has fostered a form of rejection sensitivity among Jews who often view the rest of the world as inherently hostile to them (Hirschberger et al., 2010). Today, as explicit anti-Semitism is considered unacceptable in many societies, but at the same time criticism of Israel’s policies are on the rise, it becomes increasingly difficult to differentiate legitimate opposition to questionable policies from deep-seated hate cloaked in legitimate disguise. Research suggests that although a considerable amount of anti-Israeli sentiment in Germany can be directly attributed to anti-Semitism, the majority of Israel’s critics harbor no anti-Semitic sentiments whatsoever (Kempf, 2011). One telltale sign of when virulent hatred underlies seemingly legitimate criticism is the tenor of that criticism – in a study of representative samples in 50 European countries, the use of extreme hyperbolic language against Israel was predictive of anti-Semitic motivation (Kaplan and Small, 2006). In signal detection terms (Macmillan et al., 2002), conflating well-meant criticism with hate (a false positive) over failing to spot hate when it is present (a miss) may have benefits for group survival that outweigh the costs of this paranoid outlook.

This seemingly adaptive caution, however, may develop into a post-traumatic worldview that is characterized by extreme vigilance, compulsive attention to threat that may be accompanied with inattentional blindness to positive signals from other groups, and the sense that the group is alone in this world and must fend for itself (Hirschberger et al., 2017). At the individual level, this perception of the world may cultivate anxiety and compromise achievement (Mendoza-Denton et al., 2002); at the collective level, the chronic distrust of others might foster extreme self-reliance and an aggressive stance toward any threat, big or small. If existence is capricious and the group stands alone against the entire world then any threat must be considered an existential threat as there is no margin for error and no tolerance for incorrect rejections of a threat that may turn out to be real; responses must be swift and powerful, and because life itself is at stake, the moral justification for action is incontrovertible (Hirschberger et al., 2017). This post-traumatic worldview may no longer serve the evolutionary adaptive function of protecting the survival of the group and may, ironically, compromise the safety of group members by favoring aggressive policies that may not always be required, and that may propel the group into unnecessary conflict. Why then do group members cling to seemingly detrimental worldviews that may not serve their best interests?

### Trauma Is of Death, and Death Creates Meaning

Although the colloquial use of the term *trauma* often refers to relatively benign events (“my visit to the dentist was traumatic”), the psychological definition of trauma includes the encounter with death, or extreme death anxiety, as a central component of this psychological phenomenon (Galea et al., 2003; Smelser, 2004). It is prudent, therefore, to understand the role of death in collective trauma and its relationship to the construction of meaning if we are to disambiguate the motivations underlying the resolve to perpetuate the memory of collective trauma, in spite of the detrimental effects this memory may have.

In the *Birth and Death of Meaning*, cultural anthropologist Ernest Becker asserts that: “…society is responsible, largely, for shaping people, for giving them opportunities for unfolding more freely and more unafraid. But this unfolding is confused and complicated by man’s basic animal fears: by his deep and indelible anxieties about his own impotence and death, and his fear of being overwhelmed and sucked up into the world and into others. All this gives his life a quality of drivenness, of underlying desperation, an obsession with the meaning of it and with his own significance as a creature” (Becker, 1975).

Becker (1973, 1975) further asserts that humans are a social animal, not just because of their evolutionary nature, but because of their fundamental need to seek meaning and significance. At the core of this quest for meaning resides death as a fundamental human problem. Unlike other mortal beings that live in a perpetual present, oblivious to their ultimate fate, humans are bestowed with a complex cognitive system that generates self-awareness; it enables us to remember our past, imagine our future, and project our self in our mind over time and space to wherever we may desire to be. This remarkable ability comes with a somewhat disconcerting side effect – the poignant awareness of the limited, transient nature of existence. According to terror management theory (TMT: e.g., Pyszczynski et al., 2015), the awareness of death in an animal instinctively motivated by self-preservation creates an impossible tension between the desire to live, and the ultimate recognition that death is inevitable, and that attempts to overcome this fate are doomed to fail. To deal with this irresolvable anxiety, humans have developed cultural worldviews – existential illusions (Greenberg, 2012) that give life meaning, significance and purpose. These worldviews cannot solve the problem of death, but they provide the comforting illusion that part of the self will persevere and survive physical death through cultural rites, symbols, and belief systems. This sense of *symbolic immortality* (Lifton, 1973) provides a semblance of continuity that the physical self fails to provide, and by doing so not only alleviates individual existential concerns, but embeds the individual into a symbolic collective entity that existed before the individual was born and will likely continue to exist long after she or he expire. Adherence to, and identification with this symbolic collective entity is, therefore, vital to the management of the terror of death, to the extent that the memory of the collective becomes one’s own memory; the aspirations of the collective become one’s own aspirations; and the pains and woes of this collective are experienced as genuine personal suffering.

From an existentialist perspective, therefore, the same forces that threaten to break a group may, ironically be elemental in making a group. Specifically, the memory of collective trauma that amplifies a sense of individual and collective existential threat prompts the search for collective meaning through adherence and identification with the group (Hirschberger et al., 2016a). This process of identification necessarily involves the construal of the group as special and unique in the world to the point that it is worthwhile and honorable to suffer and even die for the group, as proclaimed in the ancient Latin adage: *dulce et decorum est pro patria mori* (it is sweet and honorable to die for one’s country). In this process, the history of the group, and its traumatic past in particular become an indispensable vehicle for injecting meaning into the present struggles and confrontations of the group. The attempt to insert meaning into tragedy, and turn an otherwise pointless death into an act of heroism that corresponds with the collective memory of violence against the group, culminates at the point where the death of group members is, ironically, transformed into a symbol of group continuity and group immortality.

Suicidal terrorism, for example, is a form of wanton violence against innocent civilians wherein the terrorist sacrifices his or her own life in the process of killing random others. Some of the explanations for this seemingly irrational and senseless act suggest that a quest for meaning underlies the motivation of the suicide bomber (Kruglanski et al., 2009). By self-sacrificing for the group, the terrorist is seen as a martyr, and is transformed in the eyes of others from another unremarkable individual, part of an indistinguishable mass, into an immortal hero placed on a pedestal. This ultimate sacrifice for the group against the supposed enemies of the group not only elevates the status of the suicide bomber and grants him or her symbolic immortality at the expense of physical mortality; it connects the act to historical confrontations between the group and other groups and renders one comparable to legendary historical figures (Acosta, 2016). By doing so, the death of the young suicide bomber is transformed from an individual tragedy to a symbol of the group’s immortality, and reaffirms the connection between the sacrifices made by historical heroes and present-day martyrs.

Wars, massacres and genocide confront people with the painful realization that individual lives are extremely fragile and vulnerable, and that during violent times the value of human life is often reduced to nothing. It is at these times, in particular, that the collective self becomes invaluable; it substitutes the frustrated need for individual life with the promise that the collective will endure and survive over time. As French sociologist, Auguste Comte asserted: “The only real life is the collective life of the race [group]; individual life has no meaning except as an abstraction” (Gane, 2006). When people are confronted with massive death and with their inability to do much about it, they search for meaning and find comfort in the group – a collective symbolic structure that is greater and more enduring than the physical self (Becker, 1973), a structure that satisfies the basic elements of meaning and identity – values, efficacy, purpose and worth (Vignoles et al., 2006).

### Trauma Motivates Self-Continuity

Existential threat prompts a motivation for self-continuity and symbolic immortality through social identity (e.g., Castano et al., 2002), but what is the nature of this continuous self, and how does it serve the purpose of infusing tragedy with meaning? In recent years, social psychological theory and research have recognized the importance of the temporal dimension in social identification, and there is growing interest in the role history plays in formulating group identity (e.g., Reicher and Hopkins, 2001). Social representations theory (Moscovici, 1988; Liu and Hilton, 2005), for instance, suggests that the way people construe and explain historical events may have a marked impact on how they relate to the present, and what they expect from the future. Other accounts, suggest that the social information traveling from the past to the present comprises two main components: perceived cultural continuity – the extent to which group values and norms are transmitted from one generation to the next, and perceived historical continuity – the extent to which events in the group’s past are seen as causally interconnected and are incorporated into the group’s current identity (Sani et al., 2007, 2009).

A related conceptualization of group continuity over time makes the distinction between perceiving the group as an intra-generational entity which includes only living group members, and a *trans*-generational entity that includes all members of the group: past, present, and future (Kahn et al., 2017). A series of correlational and experimental studies conducted on Israelis, Palestinians, and Swedes demonstrates that individuals who perceive the group as *trans*-generational are more tolerant of in-group casualties that are deemed necessary to promote the group’s interests. Collective meaning, in this case, trumps the value of individual lives (Kahn et al., 2017).

This research on *trans*-generational conceptualizations of the group, highlights the distinction between the physical lives of group members and the existence of the symbolic collective in a manner that is complimentary of the existential explanations presented earlier. Namely, individuals who include past and future group members in their definition of the group are more likely to find the lives of present group members dispensable if this sacrifice is believed to promote group continuity. Collective trauma, therefore, may not only increase the desire to uphold a symbolic continuous collective self; it may shift concern from the effects of the trauma on individual group members to the implications the trauma may have on the future of the group.

The research on historical continuity and *trans*-generational identification add another layer of understanding to the role of trauma in the construction of collective meaning. If the existential anxiety emanating from trauma is a driving force behind the construction of a symbolic continuous collective self, to the extent that individual life is dispensable for the sake of group immortality (Pyszczynski et al., 2006; Routledge and Arndt, 2008), the successful construction of a *trans*-generational social identity is the pinnacle of this terror management; it enables individuals to overcome the instinctive terror that comes with exposure to the death and suffering of other group members, and instead assumes a bird’s-eye view that disregards current sacrifices, transcends the present, and envisions only the benefits the group may reap in the future (Kahn et al., 2017). Napoleon must have been in this *trans*-generational mindset when he contended that “death is nothing, but to live defeated and inglorious is to die daily.”

### Meaning Is Not Monolithic: Social Representations of Trauma

At this point of the analysis of collective trauma and meaning, we have seen how trauma creates meaning for victim groups; it alleviates existential threat, induces a search for collective meaning, operates to embed the individual in a social group that transcends physical existence, promotes a continuous historical self spanning centuries and millennia that is valued above individual life, and increases group identification and group cohesion. Societies with a history of trauma are in a constant process of constructing and reconstructing the meaning of the trauma, not so much in an attempt to understand the past, but because of a pressing need to make sense of the present. Because the present is active in shaping the memory of the past, when present conditions change the motivation to remember the past in a certain way may change as well (Rimé et al., 2015). This reconstruction of meaning constitutes weaving once again the fabric of essential connection (Baumeister and Vohs, 2002); of finding purpose, values and worth and a sense of efficacy to make a difference. These conclusions, however, assume a monolithic relationship between trauma, memory and meaning such that all group members are expected to derive the same sense of meaning from the same collective trauma. But, people understand history in different ways, and what may induce guilt in some may foster pride in others depending on how they represent the past, and on the attributions they make for their group’s wrongdoings (Doosje and Branscombe, 2003; Imhoff et al., 2017). For some, the history of genocide in Europe instills a sense of guilt and a desire to compensate for past wrongdoings by welcoming current immigration to Europe (Rees et al., 2013); for others, the same history may imply the danger of mixing with other cultures, and the need to safeguard Western civilization from unwelcome influences.

Similarly, the dictum ‘never again’ referring to the Holocaust is understood by some Israeli Jews as a call to arms to ensure that the Jewish people will never face the threat of annihilation again. For others, this same history delivers the moral imperative that Jews, having suffered the consequences of extreme racial hatred, should be at the forefront of the struggle against all forms of prejudice and discrimination, and should be especially cautious to not victimize others (Bauer, 1979; Klar et al., 2013). Thus, there appear to be individual differences in the way group members remember collective trauma and in the meaning they derive from it. Social representations theory provides a framework to understand variations in the understanding of history and how these variations impact the construction of meaning.

The study of social representations of history indicates a growing understanding that the collective representation of history does not necessarily reflect the historical truth, but rather is a combination of historical facts with shared myths and beliefs that are essential in forming and maintaining group identity (e.g., Reicher and Hopkins, 2001; Liu and Hilton, 2005). Social representations are not only based on how a group construes its’ past, but also on how other groups perceive it. Discrepancies between in-group and out-group perceptions of a group’s history, therefore, may be a source of intergroup tension.

Discrepancies may exist not only between opposing groups, but between members of the same group. Moscovici (1988) makes a distinction between hegemonic representations that are shared by most of the members of a political party, or a nation; emancipated representations – variations on hegemonic representations that are tolerated and not contentious; and polemic representations that are related to social conflicts and controversies in a society.

This distinction between consensual and non-consensual social representations is fundamental to understanding how people make sense of history, and how they understand the role played by their group and other groups. The acts of perpetrators, for instance, are often difficult to understand and both scholars and laypeople make attributions about why perpetrators acted the way they did. The roots of German behavior in WWII are a case example of polemic representations that have been discussed since the early 1940’s. Some, attributing an internal essence have described Germans as an “aggressor throughout the ages” (Hearnshaw, 1940), and as having a set of permanent characteristics that underlie their aggression: “if the criteria of a trait are permanence and lack of specificity we may rightly call aggressiveness a trait of these individuals” (Schreier, 1943, p. 211). Contemporary essentialist accounts focus on fixed worldviews and belief systems that are claimed to be uniquely characteristic of German society (Dawidowicz, 1975; Goldhagen, 1996; Imhoff et al., 2017).

Others reject the notion of an internal evil essence underlying evil acts and instead attribute wrongdoings to external forces working on the perpetrator group such as coercion by a powerful and ruthless regime. In the case of Germany, Nazi terror and fear among ordinary Germans could be offered as an alternative attribution for the horrors committed by this group (Imhoff et al., 2017). Another attribution that places the onus on the situation and not the group suggests that historical crimes are the end result of extremely harsh social and economic conditions that facilitated the rise of aggressive dictators (Imhoff et al., 2017). Historian Christopher Browning invokes the social psychological processes of conformity, compliance, and pluralistic ignorance to explain the transformation of ordinary men to mass murderers (Browning, 1992).

These different attributions underlie different representations of history, and these different representations have a profound influence on the meaning derived from the trauma. Attributing perpetrators’ behaviors to an internal, evil essence highlights the moral distinction between victim and perpetrator, and consolidates the morally superior position of the victim group; it also allows victims to avoid the uncomfortable question of whether they would have behaved similarly under similar conditions. For perpetrator groups, however, this attribution is extremely threatening; it leaves the group forever guilty of the past, with each generation carrying the burden of their ancestors’ crimes; it also forestalls any process of change, as changing the inner essence of the group is near impossible. To reconstruct a meaningful and positive group identity in the aftermath of group wrongdoings, members of perpetrator groups are motivated to attribute historical crimes to external, uncontrollable circumstances (Imhoff et al., 2017), a process that corresponds to the ultimate attribution error (Pettigrew, 1979) – a group level attribution error wherein people tend to attribute negative in-group behavior to external causes. This absolves them from the burden of guilt; allows them to draw a clear distinction between current group members and past members; and most importantly enables them to formulate a social representation of the group that isolates the dark episode as an uncharacteristic failing. In the words of Alexander Gauland, Germany’s far-right AfD leader, “Hitler and the Nazis are just bird shit on the 1000 years old successful German history.”

Social representations of history, therefore, are not merely attempts to understand what happened, but are building blocks in the construction of social identity. The intergroup animosity that existed during the trauma is often replaced with memory wars over the attributions made for the trauma and the significance of the trauma for the image of both victim and perpetrator groups. These tacit memory wars that take place between victim and perpetrator groups and within each one of these groups constitute an ongoing struggle with a troubling history and the inter- and intra-group negotiation of collective meaning.

## Part II: Perpetrators

### Trauma Threatens Meaning for Perpetrators

The manner by which an historical trauma is represented in collective memory may present an *identity threat* (Branscombe et al., 1999) to members of perpetrator groups, and may constitute a moral injury associated with loss of meaning (Litz et al., 2009). Reminding people of the responsibility of their group for past misdeeds leads to derogation (Castano and Giner-Sorolla, 2006) and to negative attitudes toward the victim group; it leads to a defensive attempt to protect the group by minimizing the historical crime (Doosje and Branscombe, 2003), distorting the memory of the event (Frijda, 1997; Dresler-Hawke, 2005; Sahdra and Ross, 2007), and justifying in-group behavior (Staub, 2006). Members of perpetrator groups often display ‘blind spots’ in their memory of the event in order to eliminate inner conflict (Frijda, 1997, p. 109; Dalton and Huang, 2014), or deny the ongoing relevance of the past by demanding historical closure on this chapter in history (Hanke et al., 2013; Imhoff et al., 2017).

The threat posed by a history of perpetration to a group’s moral image is particularly poignant for people who highly identify with their group (Ellemers et al., 2002). For them, defensive representations of history (Hirschberger et al., 2016b) are necessary to restore a positive social identity which reflects on their self-concept (Tajfel and Turner, 1979), and they are highly motivated to believe that their group is moral and good (Leach et al., 2007). At medium levels of group identification, a certain level of collective guilt seems tolerable, but high identifiers are averse to feeling guilty (Klein et al., 2011) and when reminded of their groups’ misdoings react with defensive thoughts and responses to the reprimanding source (de Hoog, 2013), feel less collective guilt than low identifiers (Doosje et al., 1998), and also increase their opposition to the out-group (Smeekes and Verkuyten, 2013).

### Denying the Trauma

The most extreme form of defense against the threat posed by collective trauma to the moral image of perpetrators is to deny that the traumatic event ever took place. If the trauma never happened, not only is the alleged perpetrator innocent of any wrongdoing, there is a complete reversal in the perpetrator-victim relationship such that the supposed victim is in fact the aggressor who is casting false allegations against others, tarnishing their reputation, and receiving undeserved reparations for harm that was never committed. In this case, the crisis to meaning facing the perpetrator group turns into an opportunity to fortify the group’s moral standing. This type of victim blaming that is common in sexual crimes (Campbell and Raja, 1999), is more difficult to sustain in cases of collective trauma because of the number of witnesses and the quantity of physical evidence that is hard to discount. Nevertheless, as time passes from the collective trauma, and the direct witnesses are gone, it becomes more challenging to confront such historical revisionism (Lipstadt, 2012).

### Reconstructing the Trauma

In the aftermath of collective trauma, discerning victims from perpetrators, and willing collaborators from collaborators at gunpoint, is often not clear cut. This ambiguity provides ample room for the reconstruction of history and the creation of a collective memory that is favorable to the group. The role played by Hungary and Poland in WWII – nations that stood on both sides of the victim-perpetrator divide – are case examples of how groups may construct a selective account of history that contains only favorable information about the group, while disregarding and distorting information that may compromise its positive image.

Whereas there is no doubt that Poles were the victims of the Nazis with three million ethnic Poles murdered during WWII, information has surfaced, in recent years, revealing horrific acts of mass murder perpetrated by Poles on their Jewish neighbors at their own volition, and not under Nazi coercion (e.g., Gross, 2001; Grabowski et al., 2013). These findings undermine the prevalent narrative of Poles as mere bystanders or victims, threaten the moral image of the group, and fuel vibrant debates in Poland about truth and memory (Bilewicz et al., 2014). Further, the attempt to defend an untarnished image of victimhood has stimulated victimhood-related anti-Semitism (Bilewicz and Stefaniak, 2013). Recently, the Polish Senate approved a controversial bill making it illegal to accuse the polish people or state of complicity in the Holocaust (John, 2018) indicating how memory wars are, not mere intellectual debates over history, but desperate attempts to salvage the image of the group and the meaning it provides in the face of a tumultuous past.

Similarly, while there is little question that many Hungarians were victims during WWII (such as members of left-wing movements and other dissidents), the historical record points to the uncomfortable fact of official and widespread Hungarian participation in the Final Solution (Stauber, 2010). Unlike Poland, however, that never officially collaborated with the Nazis, the Hungarian fascist Arrow Cross regime was an official ally of Nazi Germany (Deak, 1979). Nevertheless, many Hungarians today prefer to overlook the official collaboration of the Hungarian government, and maintain instead that Hungarians were forced to collaborate with the Nazis against their will, or were even victims of the Nazis. This defensive strategy dubbed by historians “an assault on historical memory” (Braham, 1999), is associated with both high nationalism and high antisemitism (Hirschberger et al., 2016b), indicating that the motivation to assume the coveted victim status includes competitive victimhood dynamics (Noor et al., 2012). Groups with a history of perpetration, however, do not always attempt to escape guilt, but either develop injunctive norms that suggest that they should not feel guilty (Bonnot and Krauth-Gruber, 2018), or some members of the group, such as older generations, protect themselves from negative feelings about the in-group by altering collective memory (Licata and Klein, 2010).

The debate over the role of Poland and Hungary in the Holocaust is a veritable struggle to salvage group meaning that is as poignant today as it ever was. When former FBI Director James B. Comey reflected on Hungary and Poland’s role in the Holocaust at the 2015 annual dinner of the United States Holocaust Museum, a diplomatic storm ensued with a summoning of American ambassadors, angry rejections of the allegations, and a demand for an apology. These incensed reactions over the memory of a distant past are telling of the powerful relations between trauma, memory, and current group meaning.

### Closing the Door on Trauma

Unlike third party collaborators that enjoy some degree of freedom in constructing a positive collective memory of the past that is not blatantly false, the direct perpetrators of trauma have no such luxury. They must either contend with their past, deny it, or alternatively, they may simply wish to close the door on history and never look back. Historical closure may convey benefits for both victims and perpetrators when closure is part of a reconciliation process (Hanke et al., 2013). In this case, closure may indicate a symbolic departure from the past that entails the construction of consensual memory about the conflict. When the motivation for closure stems only from the perpetrator group, however, it may reflect fatigue with the burden of blame, and may be associated with hostility toward the victim group (Imhoff et al., 2010).

Many Germans, for example, feel that they should no longer suffer for the sins of their ancestors, and resent the expectation that they should feel guilty over the Holocaust (Ahlheim and Heger, 2002; Imhoff et al., 2010). Many Jews, on the other hand, feel that the Holocaust cannot be forgiven or forgotten and expect Germans to recognize their collective responsibility (Cherfas et al., 2006). Victims of other traumas also display a greater need to remember, are more reluctant to forgive and may harbor lingering antipathies toward their former nemesis, even generations later (e.g., Olick and Levy, 1997; Pennebaker et al., 1997; Paez and Liu, 2011; Hanke et al., 2013).

These differences in temporal perspectives between perpetrators that are motivated to look forward and turn their back to the past (Hanke et al., 2013; Imhoff et al., 2017), and victims that tend to place much weight on the past as a source of identity (Kahn et al., 2017), and as a prism through which to understand the present (Hirschberger et al., 2016a, 2017 Canetti et al., 2018) reflect another dimension in the memory war between victims and perpetrators. For one side the past poses a formidable threat to meaning, and for the other, the very essence of meaning stems from the same traumatic past. In identity process terms (Vignoles et al., 2006), for perpetrator groups that dissociate from the past, the need for self-esteem trumps continuity (i.e., to feel good about the group they are motivated to sever ties with the past), but for victim groups, self-esteem is inextricably tied to continuity. An interesting study conducted in the context of the Rwandan genocide indicates that not all members of the victim group share the same motivation with regards to the past, and that direct survivors of violence experience more difficulty moving on and reconciling with the other group than in-group members that were not direct survivors (Kanazayire et al., 2014). Thus, the different lessons learned from the past are not only between victim and perpetrator groups, but within each group as well.

### Acknowledging Responsibility

One of the most difficult decisions perpetrator groups face is whether to accept responsibility for past transgressions and apologize for the harm they have done. Acknowledging responsibility may be devastating for a group’s moral image and for its sense of meaning and significance. It is no wonder, then, that many groups are reluctant to admit their faults and moral failures. This is true for Turkey and the Armenian genocide; the Japanese occupation of Manchuria and Korea; and the Palestinian Nakba during Israel’s war of independence. In all of these cases, and many others, acknowledging responsibility is costly as it requires change in the national narrative; it requires an incorporation of the victim’s narrative and the recognition that victorious moments for the group (such as achieving independence) are often accompanied by harsh transgressions toward other groups that may cast a dark shadow on these celebrated moments (Bar-Tal and Salomon, 2006; Hammack, 2011).

The literature on in-group responsibility for historical crimes reveals a complex picture with motivations to both defend the in-group and repair relationships with the out-group (Gausel et al., 2012). Members of perpetrator groups who do acknowledge the past crimes of their groups tend to have more positive attitudes toward the victim group (Čehajić-Clancy et al., 2011; Hirschberger et al., 2016b), more contact with victim group members, and a heightened ability to take the perspective of the victims (Čehajić and Brown, 2010).

There is a price, however, to such acceptance of the group’s past. Acknowledgment of in-group culpability creates dissonance between the motivation to believe that the group is good and its’ past misdeeds. As a result, the wrongdoings of a perpetrator group might lead members to disassociate themselves from the actors of the crime (Marques et al., 1988; Branscombe et al., 1993). The dissonance between the desire to view the group in a positive light and the fact of its dark history can either be resolved by assuming a defensive representation of history that vindicates the group (and secures an exclusive victim consciousness as described by Vollhardt, 2012, or feelings of competitive victimhood as in Noor et al., 2012), or alternatively by distancing from the group and defending the self from the association with a group with a negative reputation.

Although acknowledgment of responsibility for past crimes seems incompatible with current in-group identification for members of perpetrator groups (Čehajić and Brown, 2010; Hirschberger et al., 2016b), some historical perpetrators have struggled to create new meaning for their group while acknowledging, not denying the dark past. In Germany, for instance, the term *Vergangenheitsbewältigung* means the struggle to overcome the negatives of the past by raising uncomfortable questions about collective culpability and group responsibility. German churches have led way in this process and have developed a post-war theology of repentance. Similarly, the Holocaust is part of the school curriculum from elementary school onward. The current prevailing liberal attitude in Germany that favors multiculturalism and opposes militarism of any form can be understood as a new cultural identity that uses the difficult past constructively as the backdrop of its current positive image. This reconstruction of meaning through acknowledgment of past transgressions has, thus far, received only some empirical attention (e.g., Rensmann, 2004).

## Conclusion

Collective trauma is a devastating event in a group’s history that has far-reaching effects and profoundly influences both perpetrator and victim groups many years after the events have ended. Until recently, the psychological literature has focused almost exclusively on psychopathology and health-related consequences of collective trauma (e.g., Yehuda et al., 2002). But today, there is a burgeoning interest in understanding the social and political implications of perpetration and victimization as well (Vollhardt, 2012). This literature has already yielded several important insights: for example, it has demonstrated the relationship between collective victim beliefs and the justification and legitimization of current political violence (Maoz and Eidelson, 2007; Wohl and Branscombe, 2008; Vollhardt, 2012), and has also delineated the experience of collective victimhood, the material gains, and competition over these gains that are associated with it (Noor et al., 2012).

The current paper offers another perspective that is based neither on pathology nor on the belligerent consequences of trauma. Instead, it views collective trauma as a genuine experience with real consequences for subsequent generations. The preponderance of literature on historical victimization is situated in the intergroup relations literature (Noor et al., 2017), and is influenced by the goals and the central assumptions of this literature. Because one of the core goals of intergroup relations research is to understand and promote conflict resolution and reconciliation, the long-term effects of collective trauma are often evaluated by this criteria. Accordingly, historical victimization is typically understood as a barrier to peacemaking and a distorted lens (Schori-Eyal et al., 2017). In this paper I contend that the memory of victimization has both adaptive and maladaptive manifestations. Although members of victim groups may be less trusting of adversaries and more reluctant to compromise and make peace, this reaction may, at times, protect the group from duplicitous gestures of peace from disingenuous adversaries. Although the memory of trauma may foster a paranoid and paralyzing post-traumatic outlook, it may also spur growth through the meaning derived from the trauma. A meaning that emphasizes the resilience of the group and its ability to rehabilitate and change in the aftermath of calamity. These consequences are especially pertinent as these new generations of victim and perpetrator descendants attempt to construct social meaning that can explain the past, provide a roadmap to navigate present challenges, and prepare the group for the future.

## Author Contributions

The author confirms being the sole contributor of this work and approved it for publication.

## Conflict of Interest Statement

The author declares that the research was conducted in the absence of any commercial or financial relationships that could be construed as a potential conflict of interest.
